# Primary Pediatric Primitive Neuroectodermal Tumor of Kidney Presenting as a Lumbar Abscess: A Rare Case Report

**DOI:** 10.15586/jkc.v13i3.483

**Published:** 2026-07-15

**Authors:** Shatakshee Tewari, Varsha Kumar, Vatsala Misra

**Affiliations:** 1 Department of Pathology, Autonomous State Medical College, Hardoi, Uttar Pradesh, India;; 2 Department of Pathology, Moti Lal Nehru Medical College, Prayagraj, Uttar Pradesh, India

**Keywords:** Child, Cytology, Diagnosis, Pnet, Renal

## Abstract

Renal sarcomas are rare, accounting for less than 1% of all renal malignancies. Ewing sarcoma/PNET of the kidney is an aggressive and extremely rare neoplasm with only 120 cases reported so far. It is seen in young adults, and only few pediatric cases have been reported so far. We report an 8-year-old boy presenting with progressive left lumbar swelling for 1 year and a prior history of antitubercular therapy. Examination revealed a tender subcutaneous abscess measuring 7×5 cm in the left lumbar region. Fine-needle aspiration cytology suggested a small round blue cell tumor. Histopathological examination, supported by immunohistochemistry, confirmed the diagnosis of renal PNET, which is highly aggressive as compared to PNET arising from other sites. It needs to be distinguished from other primary renal tumors owing to its poor prognosis and aggressive nature. Clinical and radiographic features are nonspecific leading to diagnostic challenges. Definitive diagnosis requires histopathological examination and IHC.

## Introduction

Ewing sarcoma family tumors (ESFTs) were historically categorized as primitive neuroectodermal tumor, Ewing sarcoma, and Askin tumor but are now considered a single group of tumors unified by a common genetic mutation. They are a group of aggressive malignancies with characteristic EWS–FLI1 fusion gene formed due to translocation between chromosomes 11 and 22 (1). ESFTs predominantly affect children and young adults. They represent 1% of all sarcomas and most frequently arise from bone and deep soft tissues (2,3). Primary involvement of the kidney is exceedingly uncommon, accounting for less than 1% of all renal malignancies (4).

Primitive neuroectodermal tumor is a highly malignant small round cell neoplasm thought to originate from neural crest cells (5). It was first described in the ulnar nerve in 1918 by Arthur Purdy Stout. Later on, it was included in the family of small round cell tumors and named as Ewing’s sarcoma by James Ewing in 1921 (6). The morphological, immunohistochemical, and genetic features of PNET are highly comparable to Ewing’s Sarcoma (7). Although commonly encountered in osseous and paraspinal locations, it has been reported in several extraosseous sites including the chest wall, extremities, central nervous system, and rarely, the genitourinary tract (3). In rare instances, it can also present as primary renal mass.

Renal PNET was first reported by Mor and colleagues (8). It is considered a highly aggressive tumor and is often associated with rapid progression and an unfavorable prognosis compared with similar tumors arising at other sites (9). Because of its rarity and nonspecific clinical and radiological features, diagnosis can be challenging and often requires correlation of histopathological findings with immunohistochemical and molecular studies. Herein, we report a rare case of primary renal PNET presenting as a lumbar abscess in a young boy and discuss its clinicopathological features along with a review of the literature.

## Case Report

**Figure 1:**
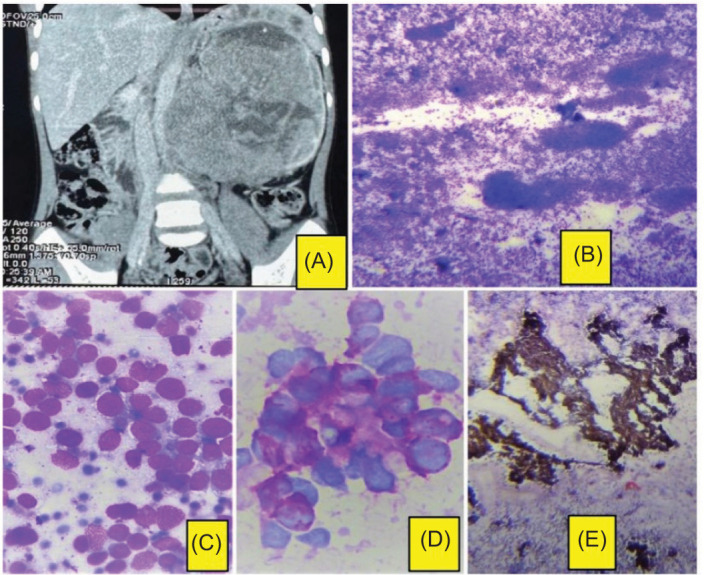
(A) CT Triphasic angiography shows large, ill-defined heterogeneously enhancing mass arising from the left kidney. (B–E) FNAC-highly cellular smears of atypical cells (×40). Dual population of atypical cells; cytoplasmic vacuolation in large cells (×400). Areas of calcification seen (×100). PAS positive tumor cells seen (×400).

An 8-year-old male child came with left loin swelling for 1 year, which was progressive in nature and was associated with mild pain. The patient had taken ATT 1 year back. Routine blood investigations indicated mild anemia and leucocytosis. On examination, a subcutaneous swelling was present in the left lumbar region measuring 7 × 5 cm in size, which was firm in consistency, inflamed, and tender. A provisional diagnosis of lumbar abscess was considered. Informed consent was obtained, and FNAC was done which yielded pus. Smears prepared were highly cellular and showed atypical cells scattered diffusely. These atypical cells were small to medium in size. Two distinct cell populations were noted: large cells with pale chromatin and scant cytoplasm with vacuolation and small darkly stained cells. The cytoplasm showed PAS positivity. Provisional diagnosis of small round blue cell tumor favoring primitive neuroectodermal tumor was made. CT triphasic angiography was done and was suggestive of large ill-defined mass with cystic and necrotic areas, likely Wilms tumor (Figure 1).

**Figure 2:**
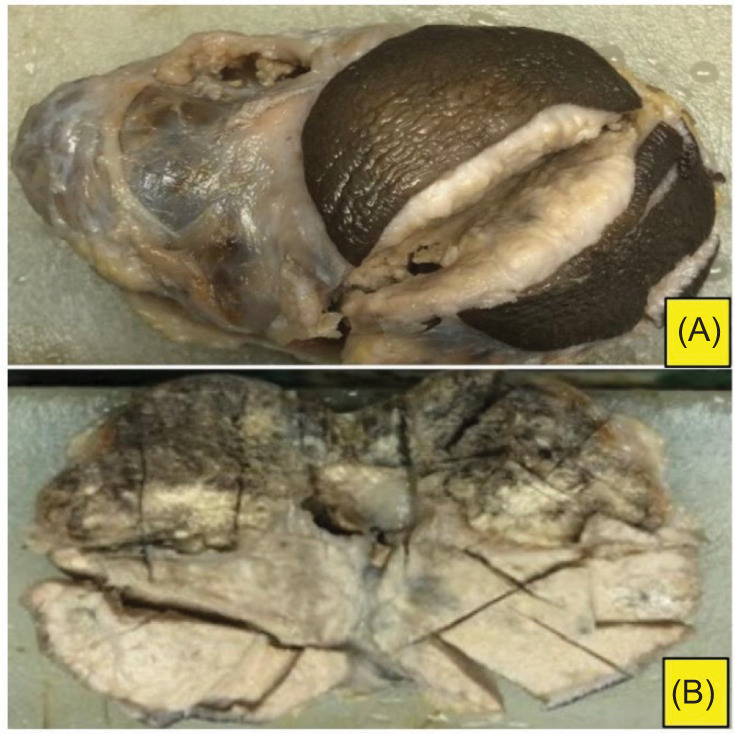
(A) Gross appearance shows tan colored renal mass with a fibrous capsule. (B) Cut surface of the mass shows absence of corticomedullary differentiation with large areas of necrosis.

**Figure 3:**
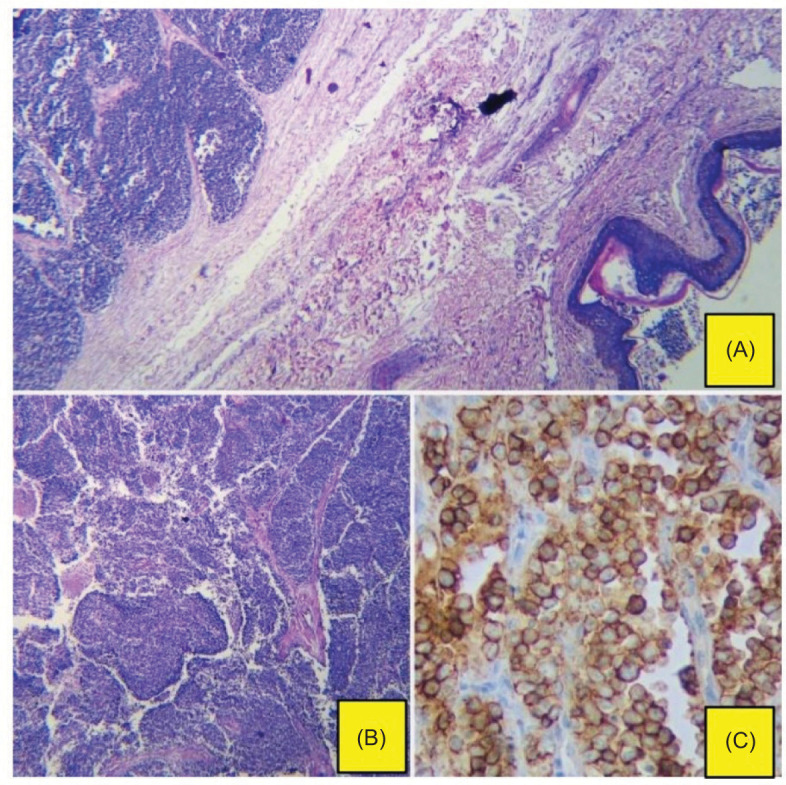
(A) Nests of tumor cells divided by fibrous septa into lobules (H&E, ×40). (B) Tumor cells seen in the subepithelial zone (H&E, ×40). (C) Tumor cells showing CD99 positivity.

Nephrectomy was performed (Figure 2) and was submitted for histopathological examination. On histopathology, nests of monomorphic round tumor cells were seen divided by fibrous septa into lobules. These cells were small, round, displayed high N:C ratio and finely stippled chromatin. No rosettes were seen. Areas of necrosis, calcification, and tumor emboli were noted. Mitotic activity was not significantly high. Tumor cells displayed positivity for CD99 (Figure 3) and were immunonegative for Myo D1. Hence, the diagnosis of a renal PNET was confirmed.

## Discussion

PNET/ES are exceedingly rare and constitute 1% of all sarcomas (2,3,10). It is a malignant small round cell tumor and is of neural crest origin (11). Researchers such as Parham et al. propose that primary renal PNETs might develop from celiac plexus adrenergic nerve fibers that penetrate the kidney. Alternatively, they hypothesize that embryonic neural crest cells could migrate into the renal tissue during development and subsequently undergo malignant transformation (12,13). Commonly, PNETs arise in the ribs and paraspinal areas. The involvement of skin, soft tissue, lungs, kidney, and retroperitoneum is rare (10).

Renal PNET was first defined in 1975 by Seemayer, almost 60 years after the original description of peripheral PNET (13). The most substantial published series so far, consisting of 34 cases, was reported by Karpate et al. in 2012. Around 120 cases have been reported with very few from India (10). Demographically, these tumors predominantly affect young adults, presenting a median onset age of around 28 years, and are approximately three times more common in males than in females (14). Our patient was an 8-year-old boy.

Patients with renal PNET often present with nonspecific clinical features. Flank pain is the most frequently reported symptom, followed by hematuria and an abdominal or flank mass. Constitutional symptoms and vascular invasion, including inferior vena cava thrombosis, have also been described. Our patient presented with swelling in the left loin along with mild flank pain. Routine blood tests offer limited diagnostic value for this condition, though patients might exhibit nonspecific elevations in serum creatinine, lactate dehydrogenase (LDH), and liver transaminases (AST/ALT). These elevations do not reliably correlate with disease stage or prognosis (11,13).

Cytological smears of PNET/ES show clusters of small round cells with variable cohesiveness and irregular nuclear contour along with Ewingoid” or pale nuclear chromatin. The intracytoplasmic glycogen can produce a tigroid background in the air -dried smears. Preserved cells may show clear vacuolated cytoplasm. Necrosis, mitotic activity, and apoptotic bodies are frequently noted (15).

Histologically, the presence of pseudorosettes and cell clusters surrounding neurofibrillary material is a useful indicator of neural differentiation. Renal PNETs are composed of primitive and poorly differentiated small, round, or ovoid cells with hyperchromatic, irregular, and pleomorphic nuclei; scant cytoplasm; and poorly defined cytoplasmic borders. Mitotic figures are common. Cells with vesicular nuclei and small nucleoli may be seen in some cases. The tumor often grows in sheets, cords, nests, or finger-like projections when invasion occurs (13).

Differentiating renal PNET from other kidney tumors requires careful morphological evaluation. For instance, classic renal cell carcinomas display distinct architectural patterns that are absent in PNETs, such as the nested acini seen in clear-cell variants, the trabeculae typical of papillary tumors, or the cobblestone appearance of chromophobe RCC (13).

The differential diagnosis also includes other small round blue cell tumors of the kidney, such as Wilms tumor, clear-cell sarcoma, desmoplastic small round cell tumor, monophasic synovial sarcoma, and neuroblastoma (7). Unlike Nephroblastoma, which is common in pediatric populations, renal PNET does not exhibit epithelial structures such as glomeruloid formations or heterologous elements (13). Other mimics include neuroblastoma, which contains Homer–Wright rosettes, and various sarcomas distinguished by specific stromal or vascular patterns (7) (Table 1).

**Table 1:** Differential diagnosis of renal PNET and other small round blue cell tumors.

S.No.	Tumor type	Key jistological features	Primary IHC markers
1	Renal PNET	Pseudorosettes, neurofibrillary material, sheets/cords of small round cells.	CD99 (+), FLI-1 (+), WT-1 (-), NSE (+/-)
2	Wilms tumor	Triphasic pattern (blastemal, stromal, epithelial); rudimentary tubules/glomeruli, common in younger children.	WT-1 (+), Cytokeratin (+)
3	Neuroblastoma	Homer-Wright rosettes, neuropil-rich background; common in younger children.	NSE (+), Chromogranin A (+), CD99 (-), FLI-1 (-)
4	Clear cell sarcoma	Nests of cells separated by a prominent vascularized stroma.	Cyclin D1 (+), BCOR (+), CD99 (-)
5	Desmoplastic small round cell tumor	Small round cells embedded in a dense, abundant desmoplastic stroma.	Desmin (+) (dot-like), Cytokeratin (+), WT-1 (+)
6	Monophasic synovial sarcoma	Fascicular growth, hemangiopericytoma-like vessels; lacks rosettes.	TLE1 (+), bcl-2 (+), Cytokeratin (+), CD99 (+/-)
7	Small cell carcinoma	Nuclear molding, high mitotic rate; typically seen in elderly patients.	Synaptophysin (+), Chromogranin A (+), Cytokeratin (+)

A targeted immunohistochemical panel is essential for confirmation. Renal PNET typically shows strong membranous CD99 positivity and nuclear FLI-1 expression but remains negative for WT-1. This profile, along with markers like LCA, cytokeratin, and NSE, helps differentiate PNET from lymphoma, small cell carcinoma, and neuroblastoma (7). Definitive diagnosis is further supported by identifying the (11;22) chromosomal translocation or in 5% of cases ERG mutation can be seen (10). Hence, the diagnosis of renal PNET is confirmed by histopathology followed by immunohistochemistry and is supported by the cytogenetic study (which was not done in our case).

Renal PNET appears to be a distinctive clinical entity, which is more aggressive than PNET arising at other sites (14). The 5-year disease-free survival rate in cases of PNET is approximately 50%, but the prognosis for patients with renal PNET appears worse (13). Primary PNET of the kidney often presents with locally advanced disease or metastasis in the initial diagnosis and is a highly aggressive malignant neoplasm (12,13). The treatment for renal ES/PNET includes surgery, chemotherapy, and radiation (10). The most commonly employed chemotherapeutic regimen includes drugs like vincristine, doxorubicin, and cyclophosphamide. Almost 90% of patients undergo surgery for the primary tumor (2). The role of radiotherapy is unclear, but it may be performed in locally advanced disease and in those with positive margins (14). Common sites for metastasis are regional lymph nodes, liver, and lung. Unfortunately, the clinical outlook remains grim; even with intensive, multimodal treatment regimens, long-term curative success is achieved in merely one-fifth of affected patients (10,14). In our case, the patient was lost to follow-up.

## Conclusion

Primary renal PNET is a unique and rare entity, most commonly affecting young adults. Despite multimodality treatment, this tumor is a very aggressive malignant neoplasm with a low survival rate. Moreover, renal PNETs are more aggressive than PNET at other sites. As renal PNET carries poor prognosis with early metastasis, it should always be considered in the differentials of renal mass in spite of low incidence in children. Morphology along with immunohistochemistry for CD99 with or without cytogenetic testing is essential for confirmation of the diagnosis. We reported this case due to its rarity in children, aggressive nature, and atypical clinical presentation.

## Declarations

### Compliance with Ethical Standards

- The manuscript has not been submitted to any other journal for simultaneous consideration.
- The submitted work is original and has not been published elsewhere in any form or language.
- The work submitted is a single study and not split up into several parts.
- Results are presented clearly, honestly, and without fabrication, falsification, or inappropriate data manipulation (including image-based manipulation).
- No data, text, or theories by others are presented as if they were the authors’ own.

## Consent

Informed consent was taken.

## Mandatory AI declaration

The authors declare that no AI-assisted tools were used in the preparation of this manuscript. All references have been manually verified for accuracy and relevance.
